# Supplementary material for: Are carbon emissions trading and green financial instruments synergistic? -Comprehensive quantitative research based on content analysis
Source: PLoS One. 2024 Mar 7;19(3):e0298601. doi: 10.1371/journal.pone.0298601 (PMC10919601; doi:10.1371/journal.pone.0298601)
Supplement: S1 Data — (ZIP) [file pone.0298601.s001.zip › Data/地级市Z_F工作报告.zip!/║┌┴·╜¡/╣■╢√▒⌡╩╨/└┤╫╘╣1⁄2╓┌║┼í╛┬φ┐╦╩2╛▌═°í┐_2018.docx]

各位代表：

　　现在，我代表市人民政府向大会报告工作，请予审议，并请市政协各位委员提出意见。

　　一、2017年工作简要回顾

　　过去一年，是新一届政府的开局之年，是持续调结构转动能提质量、增强城市竞争力的关键一年。面对艰巨繁重的改革发展稳定任务，我们在省委、省政府和市委的正确领导下，全面贯彻落实党中央“五位一体”总体布局、“四个全面”战略布局，全面贯彻落实习近平总书记对龙江两次重要讲话精神，全面贯彻落实习近平新时代中国特色社会主义思想和党的十九大精神，紧紧依靠全市人民，迎难而上，真抓实干，开创了哈尔滨经济社会发展新局面。

　　——经济保持平稳增长。去年前三季度，完成地区生产总值4159.3亿元，比上年同期增长6.6%。截至11月底，完成固定资产投资4050.4亿元、社会消费品零售总额3560.9亿元，分别增长6.6%和7.8%。全年完成一般公共预算收入368.1亿元，增长8.4%，税收占财政收入比重达到80.2%。经济发展的稳定性和协调性进一步增强，质量和效益不断提升。

　　——动能转换步伐加快。1-11月份，开复工亿元以上产业项目359个，完成投资501.2亿元，长安福特汽车、万达文旅城、波塞冬海洋王国等一批大项目投产运营。大数据、机器人、石墨烯、钛合金等新兴产业加快发展，战略性新兴产业占规模以上工业比重提高1.3个百分点。金融、文化、物流等支柱产业作用持续增强，成为支撑经济增长的重要力量。“互联网+”等新业态新模式蓬勃兴起，限额以上网上零售额增长21.3%。先进制造业和现代服务业双轮驱动的产业格局初步形成，经济发展后劲不断增强。

　　——发展活力持续释放。截至11月底，工业贷款余额超过1367亿元，增长12.1%，增速提高5.8个百分点。新增各类市场主体11.6万户，其中私营和外资企业分别增长21.9%和39.4%，带动民间投资增长13%。新增科技型企业1146户，哈尔滨新区、哈工大获批国家双创示范基地。冰雪节、迷人哈夏、中俄博览会等品牌活动和马拉松、F1摩托艇等国际赛事精彩纷呈。太平机场被国家规划为国际航空枢纽，旅客吞吐量超过1800万人次，稳居东北四大机场首位。旅游总收入超过1100亿元，增长13.2%，荣登中国十佳冰雪旅游城市榜首。哈尔滨城市影响力加速攀升，对发展要素的集聚作用不断彰显。

　　——人民生活继续改善。前三季度，城乡居民人均可支配收入达到25891.2元和10469.3元，分别增长6.8%和6.7%。全年新增城镇就业11万人，城镇登记失业率控制在3.68%，哈尔滨就业地图荣获全国创业就业展示交流活动优秀项目奖。现行标准下，47个村脱贫出列、13147人实现脱贫，贫困发生率由1.2%下降到0.98%。向市民承诺的70项惠民行动项目均已兑现，人民群众的获得感和幸福感有了新提升。

　　——精神文明、民主法治和社会治理取得新成果。市民文明素质和城市文化软实力持续提高，蝉联全国文明城市，当选2018年“东亚文化之都”。自觉接受人大法律监督和政协民主监督，人大代表建议、政协提案办复率达到100%。“七五”普法扎实开展。公共安全形势持续好转，市民对食品安全满意率由36%提高到60%，安全生产事故大幅下降，三棵树港区重大消防安全隐患整治全面完成，刑事案件万人发案率连续五年保持副省级城市最低，首获全国综治领域最高荣誉“长安杯”，社会总体保持安定有序。

　　各位代表，过去一年，我们重点推进了以下工作：

　　（一）推动产业优化升级，进一步夯实全面振兴发展的基础。坚持以千亿级产业振兴工程为牵动，推动产业发展提质增效。一是完善顶层设计。依托资源禀赋和产业基础，调整优化区县（市）产业发展重点和规划布局，制定出台了生物、通用航空全产业链行动方案和配套政策，形成了系统的产业培育新模式。二是坚持创新驱动。强化资金引导、股权激励等措施，充分发挥市校科技合作平台作用，转化科技成果379项，世界领先的哈锅燃用准东煤超（超）临界锅炉实现产业化。大力推动“双创”工作，京东、百度、腾讯旗下创业机构相继落地运营，各类科技企业孵化器发展到131家，在孵企业达到4049户，新增销售收入500万元以上科技企业246户。三是开展精准招商。重点瞄准深港、沪杭、京津等地行业龙头和领军企业，实施靶向招商、产业链招商，1-11月份，签约产业项目293个，实际使用内资1688.2亿元、外资31.1亿美元，分别增长7.1%和3.5%。四是强化要素保障。推动金融服务实体经济，投入10亿元设立产业引导基金，市企信融资担保中心为小微企业担保138亿元，新增上市、挂牌企业10户，我市成为首批国家产融合作试点城市。加强人才和数据服务保障，引进紧缺人才4639人，向社会开放800个政府数据集，数据开放水平列全国第五位。开展企业降成本行动，为企业减少税费、用地等方面成本超过90亿元。经过不懈努力，我市产业发展正步入规模扩大、结构趋优、质量提升的良性轨道，不断向着中高端水平迈进。

　　（二）统筹抓好“三农”工作，进一步壮大县域经济整体实力。深入推进农业供给侧结构性改革，促进三次产业融合发展，加快释放农业农村发展潜能。一是大力发展县域工业。坚持以“粮头食尾”“农头工尾”为抓手推进产业强县，方正鹤旭大豆深加工、宾县松杉实木家具等项目建成投产，龙头企业发展到200家，以资源加工为支柱的县域特色产业格局初具规模。二是加快建设现代农业。新建续建松花江沿岸“旱改水”工程4处、现代农业产业园31个、“两牛一猪”示范场65个，粮食和肉蛋奶总产量分别达到330亿斤和280万吨。强化市场营销和品牌培育，自建农产品电商平台实现区县（市）全覆盖，五常大米以640亿元的品牌价值位列全国大米类榜首，我市获得“中国优质稻米之都”称号。三是扎实推进农村综合改革。半数以上行政村完成清产核资，阿城区土地确权登记和方正县农村集体资产股份权能改革试点成为全国示范。新增农民合作社规范社120个，规模经营面积扩大到1800万亩，现代农业经营体系逐步完善。四是持续推进新型城镇化和美丽乡村建设。推动10个国家和省级重点镇提档升级，新建美丽乡村示范村120个。建成通车农村公路367公里，改造公路危桥25座、危房1.2万户，解决21.31万人饮水安全问题。经过不懈努力，我市农业发展方式和农村环境面貌发生可喜变化，为全面建成小康社会打下了坚实基础。

　　（三）全面深化改革开放，进一步增强经济发展的内生动力和活力。把改革开放作为推动发展的关键一招，加快构建统一有序、公平竞争的市场环境。一是推进行政体制改革。精简市本级行政权力816项，商事登记“负面清单”改革在全省推广，行政审批效率进一步提高。群众热切关注的不动产登记交易实现一体化管理、一站式集成服务。积极推进事业单位改革，所有与市直部门相关的行业商会、协会全部实现脱钩。二是推进国资国企改革。物业供热和哈投集团完成合并，首批60户市属国企面向社会推进混合所有制改革，电碳厂与法国美尔森完成重组。实行以业绩为导向的出资企业负责人分类考核及薪酬管理办法。清理退出僵尸企业11户，厂办大集体改革补漏基本完成。三是推进新区改革发展。率先在全省实现行政审批“三张清单一张网”，在区街一体化管理、集中行使行政执法权、雇员制选聘人才等方面闯出了新路。新区产业集聚功能日益增强，万鑫石墨导电浆料、百威英博二期等项目相继建成，松北科技创新、平房现代制造和呼兰大健康产业呈现出集群发展态势。四是推进深度开放合作。新开通符拉迪沃斯托克—哈尔滨—曼谷第五航权航线，进一步完善中俄本币结算、在线支付功能，1-11月份，哈俄哈欧班列、对俄电商小包发运货值分别达到7.5和8.08亿美元，实现对俄进出口额增长128.3%。综合保税区封关运行，临空经济区飞机循环再制造项目达到运营条件。深哈对口合作实现良好开局，保利东北亚文化综合体、天恒山滑雪场等20个合作项目取得实质性进展。经过不懈努力，向改革要活力、向开放拓空间的发展态势正在形成，哈尔滨正以更加自信和积极的姿态走向未来。

　　（四）持续加强城市规划建设管理，进一步提升城市综合承载力和吸引力。围绕产业发展和民生需求，进一步强化基础设施建设，补短板，强弱项，推动城市管理提档升级。一是突出规划引领作用。调整土地利用总体规划，启动中心城区多规合一，完成哈尔滨新区总体规划和综合交通、供热等专项规划，以及群力西区、新一地区等20余项城市设计。二是完善城市载体功能。新建改造道路73条、桥涵15座，新建续建各类源网厂站21座、地下管网80公里，地铁3号线一期载客运营，哈站北站房正式投用，职工街打通工程、一面街地道桥竣工通车，松花江水源工程具备供水能力，地下综合管廊建设和“公交都市”创建持续推进。三是提升城市管理水平。全面开展“美丽城区”创建，规范居民小区架空线缆6500余处，清理垃圾1.3万余吨，拆除各类违法建筑181万平方米。为团结、平山等21个镇配备环卫车辆，推动机械化作业向城郊延伸。被国家旅游局评为全国厕所革命优秀城市。开展交通大整治，动态、静态交通双管齐下、综合治理，严厉打击疯狂渣土车，取缔黑出租等非法营运车辆335台，查处各类交通违法行为超过600万件，主城区交通状况有所改善。四是治理城市生态环境。超额完成省下达的黄标车淘汰任务，投入14.7亿元，改造、淘汰燃煤锅炉2359台，市区10蒸吨及以下燃煤小锅炉基本退出供热市场。餐厨垃圾资源化利用和无害化处理项目建成投用，黑臭水体和阿什河治理取得积极进展，中央环保督察组反馈的21项年度整改任务基本完成。人工造林16.4万亩，城区新植树木14.8万株。经过不懈努力，哈尔滨变得越来越干净、越来越有序、越来越有魅力，正向着现代化国际化城市目标阔步前行。

　　（五）着力保障和改善民生，努力为全市人民办实事解难题。市财政用于民生领域支出占全部支出70%以上，各项民生任务得到落实。一是提高住房和社保水平。开工建设各类保障房8256套，为2.1万户困难群众提供住房保障，维修老旧住宅73万平方米，25.8万户居民不动产登记历史遗留问题得到妥善解决。企事业离退休人员基本养老金和城乡低保标准稳步提高，城乡居民大病医疗保险实现市级统筹，部分跨省异地就医实现直接结算。发展医养结合、旅居养老等新模式，12349公益平台集聚助老服务组织300余家，成为中央财政支持居家和社区养老服务试点城市。二是扩大教育和医疗服务供给。新建改造中小学校28所，义务教育集团联盟成员发展到400所，组建市医药、食品两个职业教育集团。开展师德师风建设年活动，乱办班、乱补课、乱收费现象得到遏制。142家市区公立医院全部取消药品加成。建成医联体79个，分级诊疗体系初步形成，共乐、建国和通江社区卫生服务中心跻身全国百强。三是努力满足群众文体生活需要。推出《祖宾梅塔与以色列爱乐乐团交响音乐会》《天鹅湖》等高雅艺术演出1000余场，举办国家级、世界级体育赛事20余项。群力外滩湿地公园、中东铁路印象馆建成开放。首届社区文化艺术节、全民健身展示周等群众性文体活动蓬勃开展，群众精神文化生活更加丰富多彩。民族宗教、外事侨务、双拥和预备役、统计、审计、信访、气象、人防、地震、档案、地方志、残疾人事业等工作取得新成效。经过不懈努力，一些困扰群众多年的现实问题得到有效解决，全市人民分享到了更多改革发展成果。

　　（六）狠抓政风建设，进一步提高服务经济社会发展的能力和水平。扎实推进“两学一做”学习教育常态化制度化，厉行法治，严格管理。一是开展作风整顿。聚焦“三个坏把式”“五个坏作风”，以严厉问责倒逼作风转变，查处不作为乱作为等破坏经济发展环境案件108起，问责282人，对广大公职人员起到了有效的警示教育作用。二是加强诚信建设。以政府诚信带动社会诚信，突出抓好政府违约问题专项整治，督促政府部门和区县（市）履约践诺，相继返还拖欠企业款4.1亿元。启动实施失信被执行人、房地产开发违规等“黑名单”联合惩戒机制，推动我市在国家城市信用监测排名大幅提升。三是强化社会监督和督办落实。通过政务公开、企业评议等途径，推动政府部门提升服务质量。对重要事项实行全程跟踪问效，市政府重点督办的1450个事项基本完成。经过不懈努力，广大公职人员责任意识和干事劲头得到新提升，为进一步提高行政效能提供了有力保障。

　　一年来，我们深切体会到，面对经济发展新常态带来的压力和挑战，哈尔滨之所以能够保持经济平稳增长、社会和谐进步，关键在于我们更加坚定自觉地践行习近平新时代中国特色社会主义思想，推动党中央治国理政新理念新思想新战略在哈尔滨落地生根；关键在于我们始终坚决贯彻落实省委省政府和市委重大决策部署，充分发挥省会城市龙头带动作用，奋力在全省振兴发展中干在实处、走在前列；关键在于我们始终坚持新发展理念，准确把握发展的阶段性特征和普遍性规律，推动经济社会各项事业向更高层次发展；关键在于我们始终坚持问题导向，面对矛盾不回避、遇到困难不退缩，勇于破除制约发展的体制机制障碍，持续释放改革红利；关键在于我们始终坚持以人民为中心，保持永不懈怠、锲而不舍的拼搏劲头，持之以恒转作风、优环境、惠民生，办成了许多百姓关心的身边事，努力提升政府公信力和城市凝聚力。这些成绩的取得，是党中央国务院、省委省政府和市委坚强领导的结果，是全市人民共同奋斗的结果。在此，我代表市人民政府，向人大代表、政协委员和社会各界人士，向中省直单位和驻哈解放军指战员、武警官兵，向所有关心支持哈尔滨振兴发展的同志们、朋友们，致以崇高的敬意和衷心的感谢!

　　在肯定成绩的同时，我们也清醒认识到，前进中仍面临着不少困难和挑战。主要是：发展不平衡不充分问题比较突出，经济稳中向好的基础还不牢固，实体经济、民营经济发展水平不高，县域经济还比较弱小，创新要素向新产业新业态新模式集聚步伐不快，旧动能减弱和新动能不足的双重压力仍然较大；公共资源配置市场化竞争不够充分，混合所有制改革进展缓慢，对外开放的功能优势还没有转化为经济优势；民生保障和社会治理能力有待进一步增强，交通拥堵、环境污染、安全隐患等现实问题仍然比较突出；部分公职人员群众观念、纪律意识、法律意识和服务意识不强，在推进改革等工作中思想不够解放，存在不严、不细、不实、不担当等庸政懒政怠政倾向，“四风”问题还有不同表现，损害群众利益和违纪违法行为仍时有发生。对这些问题，我们要下更大力气加以解决。

　　二、2018年主要任务

　　今年是贯彻落实党的十九大精神开局之年，是改革开放40周年，是决胜全面建成小康社会、实施“十三五”规划承上启下的关键一年。刚刚胜利闭幕的市委十四届三次全会，围绕贯彻落实党的十九大和省委十二届二次全会精神，提出了2018年全市工作要求，明确了发展目标、战略任务和重大举措，我们要齐心协力、认真抓好贯彻落实。进入新时代，步入新时期，我们要看到经过多年不懈努力，哈尔滨的经济基础更加坚实、民生福祉更有保障、城市发展更具活力，厚积薄发、加快发展的态势正在形成；要看到在我国经济已转向高质量发展阶段的大背景下，我市科技创新、绿色食品、大美生态、旅游文化等优势日益彰显，用资源换产业、依增量促升级的潜能正在加速释放；要看到党的十九大提出加快东北等老工业基地振兴、实施乡村振兴战略等重大举措，省委省政府作出支持省会城市建设提档升级的战略部署，为我市加快动能转换、激发经济活力、提升整体竞争力带来重大发展机遇。只要我们按照党的十九大擘画的建设现代化强国宏伟蓝图，坚持把习近平新时代中国特色社会主义思想的伟大真理力量，转化为哈尔滨经济社会发展的生动实践，只要我们充分依托现有基础，发掘潜在优势，抓住难得机遇，冲破困难挑战，聚焦中心任务，持续用功发力，就一定能够胜利实现全面振兴发展、全市人民过上更加幸福美好生活的奋斗目标！

　　今年政府工作的指导思想是：全面贯彻落实党的十九大精神，以习近平新时代中国特色社会主义思想为指导，深入落实中央经济工作会议和省第十二次党代会及省委十二届二次全会决策部署，坚持稳中求进工作总基调，坚持新发展理念，紧扣我国社会主要矛盾变化，按照高质量发展的要求，统筹推进“五位一体”总体布局和协调推进“四个全面”战略布局，坚持以供给侧结构性改革为主线，统筹推进稳增长、促改革、调结构、惠民生、防风险各项工作，推动质量变革、效率变革、动力变革，突出抓重点、补短板、强弱项，在打好转方式调结构、精准脱贫、重要领域改革、污染防治、防范化解重大风险、整顿作风优化发展环境、省会城市建设提档升级七个攻坚战方面取得扎实进展，更好发挥哈尔滨对全省的辐射功能、外溢效应和带动作用，在建设现代化新龙江征程中干在实处、走在前列。

　　主要预期目标是：地区生产总值增长7%左右，城乡居民人均可支配收入与GDP同步增长，一般公共预算收入增长7%以上，城镇登记失业率控制在4.3%以内，万元GDP综合能耗下降3.17%。着重做好以下工作：

　　（一）以提高供给质量为主攻方向，着力打造现代产业新体系。深入落实习近平新时代中国特色社会主义经济思想，坚持质量第一、效益优先，一产抓融合、二产抓提升、三产抓拓展，不断增强实体经济竞争力。一是加快推进新型工业化。做大做强千亿级食品产业，做优做精装备制造业，加速发展新材料、“大智移云”新一代信息技术等战略性新兴产业，突出抓好益海嘉里粮油、东安6AT变速箱、中联通数据中心、万鑫石墨防火涂料等项目建设。全力落实生物、通用航空产业发展三年行动计划，加快引进建设中药配方生产基地、派斯菲科生物制药扩建等项目，力争在通航技术研究、飞行器制造、运营服务保障等方面取得更大突破。抢抓国家推进工业互联网建设机遇，建成开放工业云服务共享平台，集聚全产业链企业300家以上，有效提升制造业本地配套率和全要素生产率。积极争创“中国制造2025”国家级示范区和全国质量强市示范城市。二是大力培育生产性服务业。进一步提高金融服务实体经济质量和效率，引进平安银行等金融机构，以开展“广深基金哈尔滨行”等活动为契机，引入和壮大天使投资、风险投资、创业投资，发展壮大哈尔滨股权交易中心、创新金融产业园、科技金融产业园，推进万象新天地科技金融中心等项目建设，加大对高成长科技型企业上市服务力度，切实提高企业在资本市场直接融资比重。加快发展现代物流、商贸会展、电子商务等生产性服务业，突出抓好华南城商贸物流园、空港跨境电商物流园等项目建设，重点在楼宇经济、地铁经济、共享经济、科技服务、现代供应链、人力资本服务等领域培育新增长点。加速哈港地区综合开发和高端服务业培育，打造沿江新的经济带。三是推动旅游文化体育融合发展。巩固提升千亿级旅游业，制定出台融合发展三年行动计划，推动冰雪、音乐、生态、欧陆风情等特色资源集成化、产品化。加快发展全域旅游、特色旅游和乡村旅游，着力培育“拇指文化”、电子竞技、创意设计等新业态，不断提高哈尔滨大剧院、音乐厅等文娱项目运营水平，高水平承办好首届全省旅游发展大会和城市系列节庆赛事活动。进一步推动景区景点管理提档升级，着力打造全国冰雪旅游首选目的地、冰雪人才培养高地、冰雪装备研发制造基地，做大做强冰雪产业。更加注重运用新媒体、虚拟现实等新技术新方式，增强旅游营销的实效性，持续扩大冰城夏都影响力。四是提高产业招商质量。重点围绕优势产业和国企改革重组，全力开展以商招商、定点招商、产业链招商，更多引进科技含量高、投资体量大、拉动作用强、财税贡献多的产业项目，为实体产业振兴注入新动力。

　　（二）加快建设创新型城市，着力培育以科技创新企业为主要支撑的发展新动能。继续推动政产学研用深度融合，促进孵化器、加速器、产业园一体化发展，生成更多具有较强生命力和竞争力的科技型企业。一是畅通科技成果就地转化通道。全面推广与哈工大等市校合作试点经验，建立更加稳固、更具活力的合作契约机制，深化职务科技成果“三权”改革，促进科技成果资本化、产业化。进一步发挥省工研院、市食研院作用，促进东安发动机、安天科技等企业军民两用技术双向转移，落地转化科技成果300项以上。二是提高科技企业孵化水平。建立市校所企共建孵化器新机制，用好大学大所资源，完善南岗“创新创业一条街”功能，支持发电设备创业中心、博睿之星等孵化器提升专业水平。积极引入和推广深圳湾创业广场、天安云谷等发展模式，加快完善投融资对接、政策解读、创业辅导等服务功能，探索建立覆盖科技创新创业全生命周期的推进体系。三是加强双创服务保障。放宽科技立项条件，推动大学大所、初创企业、大学生等各类主体创新创业。创新科技专项资金投入方式，落实知识产权质押贷款担保、投补联动等措施，帮助科技企业解决融资难题。做大做强哈科会，切实办好创新沙龙、创新创业大赛等各类活动，让双创星火在全市形成燎原之势。四是吸引培养优秀人才。全面落实优秀人才保障政策，探索柔性引进模式，支持重点产业发展。携手在哈高校共同开展“百万学子财汇家乡智援冰城”活动，吸引、留住、用好人才，努力让哈尔滨成为各类人才竞相汇聚、施展才华、实现创新创业梦想的热土。

　　（三）全面实施乡村振兴战略，着力推动城乡一体化融合发展。按照产业兴旺、生态宜居、乡风文明、治理有效、生活富裕的总要求，科学制定推动乡村振兴的近、中、远期规划，坚持把推进农业供给侧结构性改革作为主线，促进农业增效、农民增收、农村繁荣。一是深入推进产业强县。坚持走“粮头食尾”“农头工尾”“三产融合”的产业强县之路，大力推进宾县永和蔬菜、延寿沙棘果等涉农产业项目，统筹巴木通方依产业规划布局，加快推进巴彦皮草、木兰主食加工、依兰化工等园区建设，把食品和农副产品精深加工培育成县域经济第一支柱产业。二是提高农业供给质量。坚持质量兴农、绿色兴农，重点推进双城、五常国家级现代农业示范园区和5个“北菜南运”基地县建设，加快松花江沿岸“旱改水”和木兰香磨山、依兰倭肯河等灌区配套工程建设，粮食产能稳定在320亿斤以上。加快发展以“两牛一猪一禽”为重点的质量效益型畜牧业，肉蛋奶产量保持在280万吨以上。适应个性化、小众化消费需求，因地制宜发展特色养殖、食用菌和林下经济。依托黑龙江大米网、哈尔滨电商综合服务以及各地农村电商平台，帮助和引导农民把好产品卖上好价钱。三是加快建设美丽宜居村庄。严守生态和基本农田两条红线，坚持规划先行，着力打造一批田园综合体和特色小镇。建设农村公路200公里，改造公路危桥20座、危房5000户，解决514个村屯饮水安全问题。开展农村人居环境整治三年行动，大力推进农村垃圾、污水和村容村貌整治，持续改善农村生产生活环境。四是建立健全城乡融合发展体制机制。巩固提升土地确权颁证成果，持续深化农村集体产权制度改革，促进土地向新型经营主体集中、小农户与现代农业发展有机衔接。积极争取农村土地征收、集体经营性建设用地入市、宅基地制度改革等国家试点，探索政策性支农资金折股量化到村，进一步激活农村沉睡资源。依托农业新型经营主体引入城市资本，在城市近郊发展一批以农业和民宿共享为主要特征的共享农庄，促进农民变股东、城市消费者变农业投资者。鼓励支持城镇居民下乡和农民工返乡创业，培育新型职业农民，造就一支懂农业、爱农村、带领农民致富的能人生力军。

　　（四）切实增强改革的系统性整体性协同性，着力打造有利于激发市场活力的新体制新机制。突出问题导向，以自我革新、刀刃向内的勇气和胸怀，坚决破除一切制约振兴发展的思想羁绊和体制障碍，以思想大解放推动改革大突破。一是深化行政体制改革。巩固提升政府部门大处室、权责清单和负面清单等改革成果，借鉴深圳强区放权改革经验，将区级行使效率更高、更便民并且接得住、管得好的权力下放到区，构建权责一致、重心下移、运转协调、规范高效的行政管理体系。按照要件最少、流程最优、时限最短、信息充分共享的要求，再造审批流程，实现市、区县（市）两级窗口受理一次性提示、部门流转限时办结、证件便捷化送达。行政服务事项，除必须依法现场审验的一律开通网上办理，政府部门可共享的信息一律后台验证，社区、派出所等基层单位能承接的一律就近解决，真正做到“网上办理不见面、现场办理跑一次”，让“信息多跑路、群众少跑腿”。稳步推进政事分开、事企分开、管办分离，不断增强事业单位发展活力。落实涉企收费目录清单管理，进一步加强涉审中介治理，努力降低企业制度性交易成本。深化税收征管体制改革，切实提高征管和服务水平。二是深化国资国企改革。以最大的决心、最实的举措、最快的节奏，强力推动市属国企混合所有制改革，突出抓好哈药、哈轴、哈空调等企业引资合作，年内完成绝大部分市属国企改革任务。按照引资、混改、上市的思路，提升冰雪大世界品牌的市场价值，努力闯出一条做强做优做大国有资本的新路子。推进混改后的市属企业市场化选聘经理层，探索商业类企业市场化选聘执行董事、董事长。有效处置积压库存和应收未收账款，制定出台市属国有“僵尸企业”处置方案，纳入试点的5户企业年内全部清理退出。三是深化市场化改革。坚持公开有效原则，用好30亿产业引导资金，撬动社会资本支持产业发展，切实放大财政资金的乘数效应。更加注重运用产业思维和市场手段扩大公共服务和产品供给，鼓励社会资本进入养老、医疗等领域。加强桥体广告、公交站廊等经营权转让拍卖和土地招拍挂，强化信息披露，实现充分竞争。主动作为，有效管控政府债务，防范化解金融风险。四是大力发展非公经济。激发和保护企业家精神，鼓励和支持民营企业参与城市基础设施建设、国企改制重组、环境治理等更广领域的竞争，并在市场准入、政策落实等方面一视同仁、平等对待。切实发挥政策性融资担保作用，支持金融机构开展应收账款、非上市公司股权等权益类资产质押融资业务，让金融血液顺畅流向中小微企业。

　　（五）继续扩大对外开放，着力推动形成全方位开放新优势。发挥哈尔滨地处东北亚中心区位优势，积极融入“一带一路”“中蒙俄经济走廊”建设，巩固对俄合作中心城市地位，全方位构筑对俄罗斯、欧洲、东南亚和北美的大开放新格局。一是提升互联互通和载体功能。增强哈俄、哈欧班列运力，进一步畅通“哈绥符釜”陆海联运和哈俄公路通道。开通哈尔滨至北美货运航线，积极谋划支持有实力的航空公司开通经停哈尔滨至北美客运航线。加快推进内陆港与综保区“港区一体化”和多式联运监管中心建设，积极争取肉类、粮食、木材、整车进口等口岸功能，提升通关便利化水平。抢抓太平机场被国家规划为国际航空枢纽的机遇，系统规划布局基础设施、产业项目和配套服务，着力构筑拉动外向型经济发展新舞台。二是拓展跨境产业合作。重点围绕哈俄、哈欧、哈美等通道客户群体实施靶向招商，大力引进建设一批产业链条长、创税能力强的出口加工项目。谋划综保区配套产业园，推进临空经济区申报，促进深圳普泰森手机检测及出口等项目投产运营。发挥莫斯科海外仓、叶卡捷琳堡农业示范园等境外平台作用，着力打造跨境产业链和产业集聚区。三是推进区域合作。落实深哈对口合作框架协议，推动重点签约项目尽快落地，共建深哈合作高新技术产业园，促进两地人才双向选择、双向流动、双向认可，努力实现互利共赢。落实国家哈长城市群规划和东北三省四市振兴论坛倡议，主动参与城市间汽车、机器人产业和国家级新区等务实合作，协力打造东北振兴共同体。四是促进多领域交流和经贸往来。实施外贸五年倍增计划，紧盯俄罗斯、欧美、日韩等区域市场，扩大进出口规模。建立深圳、哈尔滨与莫斯科、叶卡捷琳堡两国四地联合创新机制，推动深度合作发展。高质量办好“东亚文化之都”中国哈尔滨年、中俄博览会等活动，促进对外开放向更宽领域、更高层次拓展。

　　（六）扎实推进哈尔滨新区建设，着力打造全市最具活力的增长极。加大国家级新区先行先试力度，加快构建新区现代产业体系和治理体系。一是加快推动新区改革创新。按照省委省政府要求部署，尽快理顺新区管理体制。全面复制推广自贸区成功做法，抓好商事登记“证照分离”改革试点，精简负面清单，不断提高投资准入、准营、贸易便利化水平，组建发改、规划、市场监管等跨行业多领域集中综合执法队伍，切实做到“一枚公章管审批、一支队伍管执法”。二是加快推动新区产业集聚。更加注重产业关联性和协调性，统筹新区产业布局，加快推进中电信数据中心、哈药总厂异地改造等在建项目，突出抓好空间环境地面模拟装置国家大科学工程等新建项目，引进落地大商城市综合体等签约项目。充分运用国家级双创示范基地、服务贸易创新发展试点等政策，管好用好新区产业引导基金，打破松北、平房、呼兰三大片区行政壁垒，推动新区招商向团组式、市场化转变，促进高能级产业项目向新区优先布局，引导科技、资本、人才等要素向新区汇集，持续扩大新区产业竞争优势。三是加快提升新区载体功能。高水平搞好新区控详规划和城市设计，推进轨道交通等现代交通设施建设，进一步完善水电气热保障体系。多渠道、多形式引进优质教育、医疗资源，加快科技创新城二期建设，努力把新区建成创新引领、功能完备、动能强劲的发展高地。

　　（七）统筹抓好城市规划建设管理，着力让城市更干净更有序更舒适。深入落实省委省政府支持省会城市建设若干意见，突出以管理为中心、规划先行和功能修补相协同，打好城市建设提档升级攻坚战。一是强化规划引领。围绕打造哈长城市群和哈尔滨都市圈，完善“一江居中、两岸繁荣、沿江发展”城市框架。完成中心城区多规合一，实现规划效能最大化。进一步优化哈东、哈西及群力西区空间布局和功能品质，推动设施配套、要素集聚、产城融合。优化轨道交通规划，完善城市功能性源网规划，抓好高铁、地铁沿线站点区域规划设计和土地控制。做好国家城市设计和城市双修试点工作。二是突出功能修补。进一步打通交通堵点，畅通道路交通主动脉和微循环，同步推进地铁1号线三期、2号线一期、3号线二期建设，全力配合哈站改造、太平机场扩建等涉铁涉空工程建设。统筹推进老旧市场有序搬迁改造，进一步疏解中心城区人口、交通和安全压力。突出抓好中东部城区集中供热、松花江水源呼兰区域供水和地下综合管廊在建工程建设，扎实推进海绵城市建设，不断提升城市运行保障能力。三是加强城市管理。持续推进“美丽城区”创建，从严整治露天烧烤、市场摊区、背街背巷、居民庭院环境乱象，保持控违拆违高压态势，推进环卫作业市场化改革，强化区级综合执法，落实网格化、精细化责任，确保主城区干净有序，城乡结合部环境改善。建立垃圾专业化收运和处理体系，依靠法治和舆论力量推进生活垃圾分类进家庭、进社区、进校园、进企业、进机关，加快推进松北呼兰、双城生活垃圾焚烧发电厂和西部地区垃圾处置环保综合产业园建设，确保城区生活垃圾无害化处理率保持在91%以上、资源化利用率达到15%。四是打造智慧城市。出台智慧城市建设行动计划，整合规划、公安、城管、交管等各类数字平台，加快构建全市统一的政务云平台体系，进一步推进政府数据开放、信息共享和挖掘应用，为培育相关产业、提高城市管理水平创造条件。充分借鉴杭州“城市大脑”等智能交通经验，更多应用新一代人工智能技术，努力实现公交车科学调度、在线查询，交通管控即时反应、智能分流，让城市运行更加高效，更加便民。

　　（八）加强污染治理和环境保护，着力建设美丽生态宜居哈尔滨。牢固树立绿水青山就是金山银山的发展理念，坚持节约优先、保护优先、自然恢复为主，构建大环保新格局。一是打好污染防治攻坚战。深入开展减霾卫蓝行动，启动20蒸吨以下燃煤锅炉淘汰工作，做好高排放机动车淘汰工作，严禁秸秆露天焚烧，建立覆盖全域的秸秆收储体系，加快推进华润400万吨秸秆沼气、五常龙冶秸秆发电和100个秸秆压块燃料化项目，大幅度提高秸秆还田和综合利用水平，坚决打赢蓝天保卫战。深入开展治污净水行动，全面落实河（湖）长制，突出抓好磨盘山等饮用水源地保护，巩固提升城市内河与黑臭水体整治成果，加大对肇兰新河等污染较重流域治理力度，阿什河水体消除劣五类。深入开展保土护田行动，加大农业“三减”和永久基本农田保护力度，争取国家轮作休耕试点，完成水土流失治理面积30万亩。二是全面加强生态保护。统筹山水林田湖草系统治理，推进退耕还林还草还湿，争创首批国际湿地城市。落实保栽保活责任制，人工造林7万亩，城区新增公园10处、拓展绿地1000公顷。全面划定生态保护红线，实施生态环境负面清单管理，努力打造山清水秀、人与自然和谐共生的美好环境。三是大力推进绿色发展。加快绿色园区、工厂、产品设计和供应链建设，提升生物质发电、光伏发电、天然气等清洁能源使用比重，推行政府绿色采购，发展绿色金融，培育壮大节能环保、清洁生产、清洁能源产业。在全社会广泛开展绿色创建活动，让绿色低碳生活成为哈尔滨的新风尚。四是完善生态监管机制。制定生态文明建设目标评价考核办法，推行排污许可制度，建立市级环保巡查监察体系，严厉打击各类环境违法行为，提高研判预警和应急处置能力。进一步完善环保信用评价、信息强制披露、碳汇交易等制度，落实国家环境损害赔偿制度，加快构建政府为主导、企业为主体、社会组织和公众共同参与的环境治理体系。

　　（九）持续加大改善民生和社会治理力度，着力让改革发展成果更多更公平惠及全市人民。进一步加大民生投入，重点在幼有所育、学有所教、劳有所得、病有所医、老有所养、住有所居、弱有所扶上不断取得新进展。一是优先发展教育事业。落实立德树人根本任务，加强社会主义核心价值观和中华优秀传统文化教育，创建足球、冰雪等素质教育、特色学校120所，学生核心素养体系试点学校实现区县（市）全覆盖。调整优化城乡学校布局，新建改造中小学校18所，实施集团化办学和校长、教师轮岗交流，推动优质教育资源向农村地区和薄弱学校配置，着力解决中小学生课外负担重、“择校热”、“大班额”等突出问题，努力让孩子们享有公平而有质量的教育。推动产教融合、校企合作、工学结合，为我市产业发展培养更多知识型、技能型、创新型适用人才。办好学前教育、特殊教育、网络教育和民办教育。开展师德师风提升年活动，重拳整治校园伤害和“三乱”问题，着力打造高素质教师队伍。二是提高就业质量。坚持积极就业政策，充分运用就业地图等信息化手段，推动高校毕业生、农民工、失业人员等群体更充分就业。新增城镇就业9.8万人。三是完善社会保障体系。抓好城乡居民基本养老保险与老农保制度衔接，继续扩大助保贷款范围，将城乡居民普通门诊费用纳入医保支付范畴。完善社会救助、社会福利等制度，切实保障困难群众基本生活。加快培育医养教结合、旅居养老、高端养老社区，创建老年宜居示范社区10个，新增医养结合养老机构床位1000张，进入12349平台的养老服务组织发展到1000家以上，为老年人提供更多更好服务。四是改善居民住房条件。坚持多主体供给、多渠道保障、租购并举解决老百姓住房问题。继续加大棚户区改造力度，基本建成棚改住房8000套，回迁进户9000户，分配公租房6000套，创建物业管理示范项目30个。推进热源联通，改造老旧入户管道，强化供热监管，切实保障人民群众住上暖屋子。五是继续加强交通综合治理。细化综合交通近期建设规划，尽快启动快速路网体系建设。新增公共停车泊位7000个，住宅小区配建车位10万个以上，通过停车场智能化改造、整治私占公共停车空间等措施，提高停车资源利用率。继续依法严管严罚占道经营、乱停乱放、非法营运、超限超载等行为，持续改善交通状况。优先发展公共交通，新增更新公交车辆400台，开辟调整延伸公交线路8条，开工建设和谐大街公交首末站和香滨路综合枢纽站，为群众出行创造方便条件。六是坚决打赢脱贫攻坚战。提高政治站位，强化主体责任，围绕贫困人口对标收入标准和“两不愁、三保障”，贫困村对标贫困发生率和“三通三有”，制定实施精准扶贫脱贫三年攻坚计划。按照基础要实、数字要准、措施有力的原则，主攻产业扶贫弱项，补齐基础设施短板，注重扶贫同扶志、扶智相结合，加强扶贫对象动态管理，用足用好各类政策措施，动员全社会力量齐帮共建，坚决完成省下达的年度扶贫脱贫任务。七是推进健康哈尔滨建设。启动公立医院现代管理制度改革，坚决整治变相指定购药吃回扣等行为，切实让群众分享到医改后的红利。进一步完善分级诊疗体系，开展紧密型医联体试点建设，做实家庭医生签约服务。加强基层医疗机构软硬件建设，改善群众就医条件，督促落实村医待遇。完成市一院全科医生规范化培训基地建设和市眼科医院搬迁改造任务。继续创建国家食品安全示范城市，完善从土地到餐桌全程可追溯的食品监管体系，努力让全市人民吃得放心。加强重大疾病防控，实施中医药治未病健康工程，促进健康关口前移，不断提高人民健康水平。八是繁荣文化事业。实施文化兴市战略，巩固提升全国文明城市创建成果。抓好国家文化消费试点，面向城乡居民推出文艺演出、电影、图书等文化消费特惠活动。继续推进“731”申遗和金上京遗址保护利用工作。择址新建市图书馆、群众艺术馆，推进音乐之城博物馆、非物质文化遗产馆建设，打造“社区健身屋”60个，安装健身路径2000件。统筹谋划东北抗联、民俗、冰雪文化博物馆等项目。广泛开展高雅艺术、冰雪艺术进校园和全民上冰雪、万人徒步行等文体活动，办好第34届哈夏音乐会，更好满足群众精神文化生活需要。九是深入推进厕所革命。坚持政府主导、因地制宜、协同推进，规划建设一批、升级改造一批、引导开放一批，加快农村、旅游景区、城市棚户区、部分公共场所等厕所建设，逐步补齐这块影响人民群众生活品质的短板。十是提升社会治理水平。坚持社会化、法治化、智能化、专业化方向加强社会治理。落实安全隐患排查整治和应急处置责任，坚决防止重特大安全事故发生。按照信访工作“三到位一处理”原则，有效解决群众合理合法诉求，防止重大群体事件发生。依法打击和惩治黄赌毒黑拐骗，特别是网络犯罪、涉众型经济犯罪活动，建设更高水平的“平安哈尔滨”“诚信哈尔滨”。提高社区治理能力，加强公共法律和社会心理服务，实现政府治理和社会调节、居民自治良性互动。继续做好民族宗教、双拥和预备役、妇女儿童、法制宣传教育等方面工作，加快构筑共建共治共享的社会治理新格局。

　　三、加强政府自身建设

　　新时代赋予新使命，新使命呼唤新担当。我们要努力打造政治过硬、执行有力、务实高效、清正廉洁、人民满意的服务型政府。

　　（一）加强政治建设，牢牢把握正确的政治方向。焕发新气象、彰显新作为，必须把政治建设摆在首要位置。一是加强政治学习。坚持把学习党的十九大精神作为当前和今后一个时期首要的政治任务，要学深悟透，融会贯通，做到始终用习近平新时代中国特色社会主义思想武装头脑、凝聚共识，自觉将新理念新战略新举措落实到产业培育、新区建设、市场化改革等全面振兴的实践中，真正把党的十九大精神转化为开拓创新、奋勇前行的不竭动力。二是强化政治自觉。教育和引导政府系统广大公职人员牢固树立“四个意识”、坚定“四个自信”，在政治立场、政治方向、政治原则、政治道路上同党中央保持高度一致，坚决维护以习近平同志为核心的党中央权威和集中统一领导，始终对党忠诚、对领袖忠诚、对人民忠诚、对事业忠诚。三是践行政治使命。推进“两学一做”学习教育常态化制度化，认真开展“不忘初心、牢记使命”主题教育，不断增强广大公职人员加快振兴发展、实现宏伟目标时不我待、只争朝夕的责任感和紧迫感，特别是面对乡村振兴、国企改革、污染治理等艰巨任务，不断激发广大公职人员敢于动真碰硬、勇于攻坚克难的精气神，让广大公职人员在光荣使命面前，勇当坚定者、奋进者、搏击者，不做犹豫者、懈怠者、畏难者。

　　（二）狠抓工作落实，着力提升政府执行力。政府的一切工作必须突出实干和担当。我们要按照习近平总书记的要求，坚持说实话、谋实事、出实招、求实效，把雷厉风行和久久为功有机结合起来，以钉钉子精神做实做细做好各项工作。一是压实责任抓落实。严格政府工作目标考核和督办检查，强化政府及部门一把手的主体责任，强化层层传导压力，强化责任最后一公里的打通和落实。坚决杜绝上热下冷、推拖闪躲、责任落空、虎头蛇尾，特别是表态多调门高、行动少落实差等突出问题。二是强化问责抓落实。坚持失责必问、问责必严，聚焦“四风”问题新动向新表现，特别是对部署的工作没人管、遇到问题绕着走、对群众困难无动于衷等现象，坚持立查立办、立整立改。重点整治一线执法、服务窗口等直接面对企业和群众的领域和岗位，从严惩处吃拿卡要、不作为、乱作为、慢作为和“中梗阻”等行为，并将其暴露于光天化日之下，让那些破坏发展环境的人，颜面扫地、无立锥之处！三是心系群众抓落实。教育引导广大公职人员牢记初心使命，永葆赤子之心，真心实意为群众排忧解难。凡是群众反映强烈的问题都必须严肃认真对待，凡是损害群众利益的行为都必须坚决纠正，凡是对群众承诺的事都必须全力兑现，以言必行、行必果的实际行动取信于民、造福于民。

　　（三）加强能力素质建设，着力打造本领过硬、清正廉洁的公务员队伍。教育引导广大公职人员苦练“八项本领”，积极营造尊法守法、勤思善为、崇廉尚洁、科学履职的浓厚氛围。一是坚持依法行政。牢固树立法治观念，认真执行重大决策合法性审查、纠偏和责任追究机制，严格规范执法，不断提高法治政府建设水平。自觉接受市人大及其常委会的法律监督、工作监督和市政协的民主监督，主动接受社会监督和舆论监督，使政府各项工作始终在法治轨道上运行。二是提高专业水准。强化知识更新，克服本领恐慌，有效推动广大公职人员及时掌握新理论，熟悉新政策，了解新知识。大力弘扬专业精神，培养专业能力，切实解决公务员队伍中存在的底数不清、政策不明、业务不精等突出问题，使刻苦钻研、精益求精、追求卓越成为思想和行动自觉。三是大兴调查研究之风。坚持拜人民为师，向人民学习，放下架子、扑下身子，接地气、察实情，“身入”更要“心至”。要围绕脱贫攻坚、县域工业、非公经济、交通管理以及和百姓生活息息相关的热点难点问题，开展深入细致的调查研究，注重在一线观察、在一线体验、在一线发现问题、总结规律，在人民群众的实践中寻找解决问题的途径和办法。四是加强廉政建设。坚决把纪律和规矩挺在前面，严格执行《准则》《条例》和中央八项规定及其实施细则，严格遵守政治纪律、组织纪律、廉洁纪律、群众纪律、工作纪律、生活纪律。进一步加强公共资金、自然资源、国有资产等领域管理和审计监督，铲除滋生腐败土壤，让广大公职人员知敬畏、存戒惧、守底线，廉洁从政、干净干事，让党放心、让人民满意。

　　各位代表，决胜全面建成小康社会和全面振兴发展的宏伟蓝图已经绘就，让我们更加紧密地团结在以习近平同志为核心的党中央周围，在省委、省政府和市委的领导下，紧紧依靠全市人民，满怀激情、拼搏奉献、锐意进取、埋头苦干，为开启哈尔滨全面振兴发展新征程、谱写人民美好生活新篇章而努力奋斗！
